# On the Treatment of Pertussis, or Hooping-Cough

**Published:** 1825-09

**Authors:** Robert Venables

**Affiliations:** Physician to the Henley Dispensary, &c.


### Art. II.—*On the Treatment of Pertussis, or Hooping-Cough.* By Robert Venables, m.b. Physician to the Henley Dispensary, &c.

Every fact which tends to elucidate the nature and treatment of disease, becomes valuable as a record in medicine. This is the case because our science is so far defective, that its practice is not established upon certain and immutable principles. If we compare medicine with the more exact sciences, we shall find that, while the principles of the latter are fixed and determined, those of the former are uncertain, and even at best but hypothetical. For instance, if we have three proportionals given, we can readily find a fourth, which shall bear to the third the same relation that the second does to the first. And this proposition admits of a determined and unvariable mode of solution. But, in medicine, though we be presented with the patient, the disease, and the generally-approved remedy, we cannot always ensure the success of our practice. Hence it is that every fact in the pathology and therapeia of medicine becomes valuable, not as forming a rule of certain and undeviating application, but as constituting an accumulation of analogies, which may serve to conduct us in doubtful and difficult cases, and ultimately lead to successful results. It is a fact too well known to be insisted on here, that a remedy which has fully succeeded in curing a disease, has also failed in a number of instances of, as far as we can apprehend, a precisely similar character. Were we capable of perceiving the minutiæ constituting those nosological distinctions, which are believed or inferred, rather than proved, to exist in the same diseases as they appear in different individuals, or even in the same individual under different circumstances, medicine would then be more of a practical art, than a system of scientific reasoning and philosophical induction.

It is only upon the above principles that we can reconcile the fact of one remedy curing in one individual a disease, which had resisted its influence in another, or yielded only to some modified application of its powers. As an elucidation of these views, I shall venture to submit the history of a very severe case of hooping-cough, which occurred in my own family, and which had resisted those means which hitherto I have found generally successful in relieving the symptoms.

Case.—A little girl, eight years old, of a thin spare habit, and very delicate constitution, was, about the beginning of April last, affected with slight cough and other symptoms resembling a cold, but which were not at first much attended to. In a few days the symptoms became much more severe, and fever set in, with a very rapid pulse, so much so that it could not be numbered. Towards the end of April, the child had emaciated very much, and the fits of coughing were attended with those convulsive efforts termed *hoop*. These fits of coughing frequently came on without any very evident cause, and almost invariably followed every introduction of any thing, solid or fluid, into the stomach. The bowels were tolerably regular, but, when tardy, they were excited by a little rhubarb and infusion of senna. The cough was most troublesome and harassing, affecting the head, producing severe cephalœa, and such languor and debility as almost to bring on fainting. The respiration was extremely difficult, and performed with a mucous rattle, which indicated an increased secretion of this fluid into the air-cells of the lungs. The accumulation of this mucus always brought on the cough, and the rapidity with which it was secreted was such, that the cough frequently came on at intervals of an hour, or even less.

A cough-mixture was prescribed as follows :

℞. Mucilag. Acaciæ, Syrupi Limonum utriusque, ℥j.

Extracti Conii, gr. x.

Syrupi Papav. ʒj. M. St. coch. j. min. urgente tussi vel prout necesse sit. Admoveatur Emp. Gal. comp. sterno.

This plan was begun on the 1st of May, but very little effect seemed to be produced by the medicine on the disease. The conium was at last increased to twenty grains, but without exerting any sensible control over the symptoms or their severity. By the 18th, the child was so emaciated, that the origin, course, and insertion of the superficial muscles could be traced almost as evidently as in a subject regularly prepared by dissection for the purpose. The debility, too, was so great, that the child took to her bed, and would not get up, and, indeed, seemed incapable of making the slightest exertion.

At this period, also, an alteration took place in the character of the sputa which were expectorated after the fits of coughing. Hitherto it had been evidently mucous, swimming on the surface of the water in the basin into which the child expectorated; it was thin, and of a clear frothy appearance: by this time, however, it had assumed a completely purulent character. It was dense, heavy, tough, and of a greenish colour; it sunk in water, even hard pump water,* drawing with it the frothy mucus in which it was enveloped. The emaciation had increased to an alarming extent, being attended with great febrile irritation, moaning in the sleep, and disturbed nights. Every thing portended a very unfavourable issue.

At this juncture I was apprehensive of the effect of leeches, the child seemed so much debilitated, and worn down by disease. In these difficulties, I determined on trying the sedative effects of the hydrocyanic or prussic acid. Accordingly, on the 12th, eight minims were added to the cough-mixture already noted: this seemed to check the symptoms. On the 18th, the prussic acid was increased to twenty minims. On the 21st, the symptoms had improved so far, that the fits of coughing were less violent and less frequent: their muco-purulent character, however, still continued; and, although there was severe febrile irritation, the debility was very great; I therefore prescribed the following pills, to be taken three times a-day :

℞. Extracti Gentian, ʒss.

Sulphatis Zinci, gr. x.

Ipecacuanhæ, gr. vj.

Extracti Colocynthidis comp. gr. xij.

Sapon. gr. x. M. ft. pil. no. xviij. sumat j. ter in die.

It unluckily happened that the prussic acid was expended at this

---

* As this spring water contained lime and varions salts in solution, its specific gravity was higher (though I had not the curiosity to determine how much,) than that of distilled or rain water: hence, the specific gravity of the sputa must have greatly exceeded that of distilled water. When placed between two discs of plate-glass, it exhibited the characters of pus, as described by Dr. Young, in his "Medical Literature," and in his work on Phthisis.

period, and an interval of a few days occurred before a supply could be procured. During this interval the symptoms increased; but on the 25th the prussic acid was resumed.

June 1.—The symptoms much diminished, both in frequency and severity, and the child seems to improve, both in spirits and strength. —The prussic acid in the mixture increased to f℥ss.

4th.—Hooping has entirely ceased, and, instead of five or six convulsive fits of coughing in immediate succession, there are now only two. The child can now get up.—Perstat.

8th.—The fits have been reduced to one only at a time, with scarcely any expectoration; no fits of coughing during the night, or, if occasionally one should come on, they attack once only. The symptoms generally much relieved and improved.—Perstat.

The treatment was persevered in till the 15th, when the symptoms had completely subsided, and all medicine was suspended. The health and strength have much improved, and the patient now takes her usual exercise, and enters into her usual amusements.

25th.—Completely convalescent.

This was one of the most severe cases, not terminating fatally, that I have met with. The constitution being naturally delicate, the severity of the symptoms speedily brought on an alarming state. There are some facts, however, in this history which, upon reflection, may prove both interesting and instructive. There was a high degree of febrile irritation manifested in this case, from an early period. The fever in this instance partook more of the nervous character than of that vascular excitement which is to be reduced only by depletive measures.* When febrile or inflammatory symptoms assume almost exclusively the nervous character, active depleting measures frequently merely increase the irritation, without in the least degree controlling the severity of the febrile or inflammatory symptoms. On the contrary, they are often aggravated by these means.

I have, upon other occasions, endeavoured to point out the proper management of such cases. They cannot be affected by any active and decisive measures, unless, indeed, that they may be aggravated; but they can neither be subdued nor reduced. They are to be relieved by the *gradual*, but *permanent*, operation of remedial means. Of this description are venesections frequently repeated, local blood-lettings, blisters, issues, and setons. In this instance, venesection was out of the question; leeches appeared hazardous at the period at which their necessity could be perceived; and their utility, as well as that of

* I cannot venture to decide whether leeches, if applied at an early period, might have been serviceable. However, before the necessity became obvious, the symptoms of debility had become too well marked to encourage the hope of much benefit from their use. General blood-letting was out of the question. I even dreaded the debilitating, or rather exhausting, effects of a blister.

blisters, was, in my mind, extremely doubtful: nothing, then, remained of these means* but issues and setons.

The objection here, however, was, that their operation could not be induced in sufficient time to exert any effectual control over the symptoms. These had suddenly assumed an alarming character, and threatened suffocation, from the rapid accumulation of muco-purulent matter, or a sudden effusion into the lungs, from the violent convulsive efforts which attended the fits of coughing. The most efficient mode of treating the case, therefore, appeared to me to be the exhibition of sedatives, in such doses, and repeated at such intervals, as to keep up a constant influence upon the action of the vascular and nervous systems. Conium has been long celebrated as possessed of considerable powers in cases of the above description, but still in the present instance it did not seem to exert its usual influence; I therefore determined to try the powers of prussic acid, in assisting the conium. I must confess that I did not calculate upon experiencing so much advantage from the sedative powers of this remedy. In my mind, its efficacy has been unquestionable,† and I have no doubt, under similar circumstances, it will be found a valuable adjunct to whatever other means we may find it necessary to adopt.

I trust I shall hardly be misunderstood, nor will it be imagined, that I regard prussic acid as a specific in hooping-cough; nor, indeed, do I consider it as applicable to every case. I should no more think of entrusting the treatment of an inflammatory species of this affection solely to the powers of prussic acid, than I should think of curing a violent pneumonia or synochal fever by opium. Prussic acid is, probably, principally indicated in those temperaments which are more purely nervous, and in which great irritability is the more predominant character. However, there are, probably, few cases in which prussic acid is not admissible at some period or other of the disease, and especially after the violence of the symptoms have been sufficiently reduced by proper depletive measures. I have since had some opportunities of trying the powers of this sedative, after the institution of proper preparative means, as above noted, and I am inclined to report favourably of its use under such restrictions.

We are too apt to consider a remedy which has succeeded in a very unpromising case, as applicable in all instances of the same diseases, no matter how they may differ from each other, either in the general or individual circumstances. It is on this

* See Clinical Report, &c.

† It will be recollected that the use of the acid was suspended for a few days, in consequence of the stock having failed; and the reader will bear in his recollection, that the symptoms seemed to increase during the interval.

account, perhaps, that we hear of remedies enjoying but an ephemeral reputation, and then falling into obscurity, and frequently unmerited neglect. I am convinced, however, that this often arises from a disposition to generalisation, and from our habits of prescribing the successful medicine under every variety of character assumed by the same disease. If we were to attend more to the particular circumstances and symptoms of each individual case, and prescribe for these rather than a *generic name*,* we should hear less of disappointments in the therapeia of medicine.

The tonic pills of zinc and gentian seemed to correct the morbid action of the extreme vessels, through which pus, instead of mucus, was secreted. As this was effected, the febrile symptoms diminished, and gradually subsided altogether.

As there were three other children, one aged nearly six years, another four, and the third two years, I of course expected, as they were not separated, that they would all be affected with the same disease. However, they all partook of the cough-mixture, the composition of which I have already stated, and, although they have all had coughs, which though, both in their character and duration, they differed widely from those which are merely the consequence of a slight cold, yet none of them were attended with hooping. Some of the fits have been violent, and during the cough the face has become suffused, yet there has been nothing like a severe degree of disease. There has been no restriction whatever upon the usual intercourse; and consequently it becomes an interesting inquiry, whether the cough with which they have been affected has been regular hooping-cough?

Can prussic acid modify the effects of the contagion of per-

---

* "A patient," says Dr. Harty, "came into the clinical wards of the infirmary, with every mark of simple dysentery. I examined her: she had no symptom of fever, and it was not possible to trace the disease to contagion. The clinical professor, a gentleman of considerable merit, and for whose attentions I ought to feel grateful, visited the wards soon after, examined the patient very carefully, and in his questions was most particular relative to the Pyrexia contagiosa. Finding, however, that the former did not exist, and that there was no ground for believing in the agency of contagion, though the other marks of dysentery were all present, it would appear, by the result, that he felt himself in some dilemma how to decide between the disease and the definition; though he had no hesitation whatsoever as to the practice which he should adopt on the occasion. At a subsequent lecture he pronounced the disease to be a diarrhœa, while he said he would treat it as a dysentery. In consequence of this decision, purgatives, &c. were administered, and after a time the patient got well. Strange, that our reason should be so warped by a definition, as to induce us to call a complaint by one name, and treat it under the name of another."—*Observations on Dysentery*, &c. p. 14, 15.

It is this inaccuracy in the relation of our facts, and the want of precision in others in their application of them to practice, that leads to those disappointments which we so frequently experience, and which has given to our science the character of mere chimerical hypothesis.

tussis? or (what amounts to the same thing,) can it enable the economy so to resist, that the effects of the contagion may be so modified or subdued, that they shall be deprived of their severity? That the coughs were those of pertussis, I myself am inclined to believe. I know that the human body will resist at one period, though it may yield to the influence of contagion at another. Whatever may be the rationale, I am disposed to believe in the fact of the three cases last mentioned being genuine, but mitigated, hooping-cough. Whether this mitigation of the symptoms arose from the exhibition of the prussic acid, or was merely the consequence of a naturally less susceptible state of the system, I shall not pretend to decide, but merely propose the question as one embracing matter of interesting inquiry. It has been lately asserted that the use of belladonna renders the human frame proof against the contagion of scarlatina: surely, if this be admitted, we can easily conceive that the sedative powers of prussic acid may so modify the economy in pertussis, as to prevent those convulsive or spasmodic efforts which constitute the *hoop*.

***Henley; June 25th*, 1825.**

